# Application of FRET probes in the analysis of neuronal plasticity

**DOI:** 10.3389/fncir.2013.00163

**Published:** 2013-10-10

**Authors:** Yoshibumi Ueda, Showming Kwok, Yasunori Hayashi

**Affiliations:** ^1^Brain Science Institute, RIKENWako, Saitama, Japan; ^2^Department of Brain and Cognitive Sciences, The Picower Institute for Learning and Memory, Massachusetts Institute of TechnologyCambridge, MA, USA; ^3^Brain Science Institute, Saitama UniversitySaitama, Japan

**Keywords:** optical probes, synaptic plasticity, Förster resonance energy transfer, fluorescence lifetime imaging microscopy

## Abstract

Breakthroughs in imaging techniques and optical probes in recent years have revolutionized the field of life sciences in ways that traditional methods could never match. The spatial and temporal regulation of molecular events can now be studied with great precision. There have been several key discoveries that have made this possible. Since green fluorescent protein (GFP) was cloned in 1992, it has become the dominant tracer of proteins in living cells. Then the evolution of color variants of GFP opened the door to the application of Förster resonance energy transfer (FRET), which is now widely recognized as a powerful tool to study complicated signal transduction events and interactions between molecules. Employment of fluorescent lifetime imaging microscopy (FLIM) allows the precise detection of FRET in small subcellular structures such as dendritic spines. In this review, we provide an overview of the basic and practical aspects of FRET imaging and discuss how different FRET probes have revealed insights into the molecular mechanisms of synaptic plasticity and enabled visualization of neuronal network activity both *in vitro* and *in vivo*.

## Introduction

The brain is a highly interconnected functional network comprised of billions of neurons that communicate with each other at synapses. Throughout life, the neuronal connectivity that subserves brain function is modified and refined in an activity-dependent manner, a phenomenon termed neuronal plasticity. Plasticity mechanisms can influence neuronal function and structure through modifications at the level of synapses, dendrites and axons (Citri and Malenka, 2008; Holtmaat and Svoboda, 2009).

Different forms of plasticity are tightly regulated by a complex network of signal transduction cascades, which are the results of protein-protein interaction, posttranslational modification, subcellular translocation of proteins, protein synthesis, etc. Therefore, the temporal and spatial precision of these events is critical to support proper brain function in the developing and mature brain. The development of probes that offer spatiotemporal detection of these cellular events is vital to our ability to examine these important molecular mechanisms in biological systems. For this purpose, optical microscopic imaging enables complex and varied neuronal signals to be captured with high temporal and spatial resolution from live biological samples.

Technological advances in the past two decades have made a significant contribution to our ability to extend fluorescent imaging techniques beyond that of simple morphological analysis. One of the key developments is Förster resonance energy transfer (FRET). First reported by Förster (1946), the technique describes how energy from a “donor” fluorophore can excite an “acceptor” fluorophore, resulting in light emission from the latter. The efficiency of FRET depends on two main factors, the distance between the two fluorophores and their relative orientation. This feature enables the change in distance and angle between two fluorophores to be calculated, leading Lubert Stryer to call FRET a “molecular ruler” (Stryer, 1978). Using this property of FRET, various optical probes have been designed to detect aspects of different cellular functions *in vitro* and *in vivo*.

The sensitivity and compatibility of FRET imaging with live imaging are critical for analyzing the molecular mechanisms of neuronal circuit plasticity. In particular, much progress has been made in recent years regarding the analysis of synaptic plasticity of excitatory synapses in excitatory neurons, which are typically formed on dendritic spines (Hayashi and Majewska, 2005; Bosch and Hayashi, 2012). FRET imaging is now being applied *in vivo* and offers a unique opportunity to study how and when neurons or synapses change and which signaling events contribute to such changes in response to stimuli in the intact brain.

In this article, we will provide an overview of the basic and practical aspects of FRET imaging, summarize currently available FRET-based probes and then discuss how these probes advanced our understanding of the molecular mechanisms underlying neuronal plasticity, mainly hippocampal long-term potentiation (LTP).

### Monitoring *in situ* biochemical processes using FRET-based probes

In 1991, Tsien's group made the first attempt to image live cellular functions using FRET (Adams et al., 1991; Zhang et al., 2002). They attempted to visualize the intracellular dynamics of adenosine 3′, 5′- cyclic monophosphate (cAMP) by designing a probe based on cAMP-dependent protein kinase, in which the regulatory and catalytic subunits were labeled with fluorescein and rhodamine, respectively. Upon binding of cAMP, the regulatory subunit dissociates from the catalytic subunit, thereby eliminating FRET.

Subsequently, they also reported a voltage sensing FRET probe utilizing fluorescein-labeled lectin as a donor and oxonol, an anionic fluorescent compound, as an acceptor in living cells (Gonzalez and Tsien, 1995). At resting membrane potential, both dyes are localized on the outer leaflet of the plasma membrane and FRET occurs. Upon depolarization, negatively charged oxonol translocates to the inner leaflet of the plasma membrane and increases the distance from the donor, leading to a reduction in the efficiency of FRET.

However, FRET approaches using small molecular weight fluorescent compounds are technically demanding. For example, generation of the cAMP probe requires the cumbersome process of protein purification, *in vitro* chemical coupling with dyes and introduction into cells. The success of the oxonol-based probe largely owed to the identification of oxonol as a fluorescent molecule that travels across the plasma membrane upon a change in membrane voltage.

The emergence of genetically encoded FRET probes in the late 1990s dramatically changed the situation. This largely owes to the development and expansion of green fluorescent protein (GFP) and its color variants (Shaner et al., 2005). In a landmark study of genetically encoded FRET probes, Miyawaki et al. developed the first GFP-based calcium indicator, cameleon using cyan fluorescent protein (CFP) as a donor and yellow fluorescent protein (YFP) as an acceptor (Miyawaki et al., 1997). Cameleon consists of a calmodulin (CaM) protein fused with a M13 sequence (a 26-residue CaM binding peptide from myosin light-chain kinase), flanked by CFP and YFP. The gly-gly motif between CaM and the M13 peptide gives this probe its conformational flexibility. In the absence of calcium, CaM and the M13 sequence do not interact with each other. However, in the presence of calcium, they form a complex, which shortens the distance between the donor and acceptor fluorophores, allowing FRET to occur. Using this probe, they observed calcium dynamics in living cells and demonstrated the potential of FRET for the analysis of neuronal circuit dynamics. Since then, probes for other molecules such as cAMP, guanosine 3′, 5′- cyclic monophosphate (cGMP), and Cl^−^, small GTP-binding protein (small G-protein), phosphoinositide and signaling events e.g., phosphorylation have been developed (Table 1).

**Table 1:** **A list of genetically encoded FRET probes**.

**Classification**	**Target**	**Name of probe**	**Year**	**Probe design**	**References**
Small molecule	Calcium	Cameleon	1997	3-2	Miyawaki et al., 1997
Small molecule	Cyclic guanosine monophosphate (cGMP)	CGY, Cygnet, pGES-DE2, cGi	2000, 2001, 2006, 2013	3-1	Sato et al., 2000; Honda et al., 2001; Nikolaev et al., 2006; Thunemann et al., 2013
Small molecule	Cyclic adenosine monophosphate (cAMP)	Epac	2000, 2004	2, 3-1	Zaccolo and Pozzan, 2002; Nikolaev et al., 2004
Small molecule	Inositol trisphosphate (IP_3_)	LIBRA, Fretino, FIRE	2004, 2005, 2006	3-1	Tanimura et al., 2004; Sato et al., 2005a; Matsu-ura et al., 2006
Small molecule	Nitric oxide (NO)	NOA-1, Piccell	2005, 2006	3-1	Sato et al., 2005b, 2006b
Small molecule	Adenosine triphosphate (ATP)	A Team 1.03-nD/nA	2012	3-1	Imamura et al., 2009
Small molecule	Estrogen	SCCoR	2004	3-3	Awais et al., 2004
Small molecule	Androgen	Ficaro	2006	3-3	Awais et al., 2006
Small molecule	Glucocorticoid receptor ligands	GLUCOCOR	2007	3-3	Nishi et al., 2004; Awais et al., 2007a
Small molecule	Neurotrophic factor	ECaus	2008	3-3	Nakajima et al., 2008
Small molecule	Nuclear receptor	conpro	2007	3-2	Awais et al., 2007b
Small molecule	O-N-acetylglucosamine (O-GlcNAc)		2006	3-3	Carrillo et al., 2006
Small molecule	Vitamin A (Retinoic acid)	GEPRAS	2013	3-1	Shimozono et al., 2013
Small molecule	Molybdate	MolyProbe	2013	3-1	Nakanishi et al., 2013
Small molecule	Glutamate	FLIPE	2005	3-1	Okumoto et al., 2005
Small molecule	Zn^2+^	eCALWY-1	2009	2	Vinkenborg et al., 2009
Small molecule	Cl^−^	Clomeleon	2000	other	Kuner and Augustine, 2000
Small molecule	pH	GFpH, YFpH	2001	other	Awaji et al., 2001
Small molecule	Glucose	FLIPglu	2003	3-1	Fehr et al., 2003
Small molecule	Maltose	FLIPmal	2002	3-1	Fehr et al., 2002
Small molecule	Ribose	FLIPrib	2003	3-1	Lager et al., 2003
Kinase	Calcium/Calmodulin-dependent protein kinase II (CaMKII)	Camui α, green-Camui α, Camk2a reporter	2005, 2009, 2011, 2013	3-1	Takao et al., 2005; Lee et al., 2009; Piljic et al., 2011; Fujii et al., 2013
Kinase	Src	Srcus	2001, 2005, 2007	3-3	Ting et al., 2001; Wang et al., 2005; Hitosugi et al., 2007
Kinase	Protein kinase C (PKC)	CKAR, CY-PKCdelta	2003, 2005	3-3, 3-1	Violin et al., 2003; Braun et al., 2005
Kinase	Protein kinase D (PKD)	DKAR	2007	3-3	Kunkel et al., 2007
Kinase	Protein kinase A (PKA)	ART, AKAR	2000, 2001	3-3	Nagai et al., 2000; Zhang et al., 2001
Kinase	Abl	Picchu	2001	3-3	Ting et al., 2001
Kinase	Bcr-Abl	Bcr-Abl activity sensor	2010	3-3	Tunceroglu et al., 2010
Kinase	c-Raf	Prin-cRaf	2005	3-1	Terai and Matsuda, 2005
Kinase	PAK1	Pakabi	2009	3-1	Parrini et al., 2009
Kinase	B-raf	Prin-Braf	2006	3-1	Terai and Matsuda, 2006
Kinase	ZAP-70	ROZA	2008	3-3	Randriamampita et al., 2008
Kinase	Akt	Aktus, BKAR, Akind	2003, 2005, 2007	3-3	Sasaki et al., 2003; Kunkel et al., 2005; Calleja et al., 2007
Kinase	ERK	Miu2, Erkus, EKAR	2006, 2007, 2008	3-1, 3-3, 3-3	Fujioka et al., 2006; Sato et al., 2007; Harvey et al., 2008a
Kinase	Insulin receptor	Phocus	2002	3-3	Sato et al., 2002
Kinase	Epidermal Growth factor receptor (EGFR)		2001	3-3	Ting et al., 2001
Kinase	Ataxia telangiectasia mutated (ATM)		2007	3-3	Johnson et al., 2007
Kinase	Aurora B kinase		2008	3-3	Fuller et al., 2008
Kinase	Cyclin B1-CDK1		2010	3-3	Gavet and Pines, 2010
Kinase	Myosine light chain kinase	MLCK-FIP	2002	3-1	Chew et al., 2002
Kinase	JNK	JNKAR1, JUNKAR1EV	2010, 2011	3-3	Fosbrink et al., 2010; Komatsu et al., 2011
Kinase	RSK	Eevee-RSK	2011	3-3	Komatsu et al., 2011
Kinase	S6K	Eevee-S6K	2011	3-3	Komatsu et al., 2011
Kinase	Focal Adhesion Kinase (FAK)	CYFAK413, FERM-sensor	2008, 2009	2, 3-1	Cai et al., 2008; Papusheva et al., 2009
Kinase	PLK1		2008	3-3	Macurek et al., 2008
Kinase	SAP3K		2009	3-3	Tomida et al., 2009
Kinase	DAPK1	DAPK1(334)-F40	2011	3-1	Piljic et al., 2011
Phosphatase	Calcineurin	CaNAR1	2008, 2013	3-1	Newman and Zhang, 2008; Fujii et al., 2013
Small G-protein	Ras	Raichu-Ras, Fras	2001, 2006	3-2, 2	Yasuda et al., 2006; Mochizuki et al., 2001
Small G-protein	Rap	Raichu-Rap	2001	3-2	Mochizuki et al., 2001
Small G-protein	Rac	Raichu-Rac1	2004	3-2	Aoki et al., 2004
Small G-protein	Rab5	Raichu-Rab5	2008	3-2	Kitano et al., 2008
Small G-protein	Rho	Raichu-RhoA	2003, 2011	3-2, 2	Yoshizaki et al., 2003; Murakoshi et al., 2011
Small G-protein	Cdc42	Raichu-cdc42	2004, 2011	3-2, 2	Aoki et al., 2004; Murakoshi et al., 2011
Small G-protein	Ral	Raichu-Ral	2004	3-3	Takaya et al., 2004
Small G-protein	TC10	Raichu-TC10	2006	3-2	Kawase et al., 2006
Signal transduction	RCC1 (GEF of Ran)	CFP-RCC1-YFP	2008	3-1	Hao and Macara, 2008
Signal transduction	CrkII phosphorylation	Picchu	2001	3-1	Kurokawa et al., 2001
Signal transduction	N-WASP	Stinger	2004	3-1	Lorenz et al., 2004; Ward et al., 2004
Signal transduction	Adrenergic receptor	α_2A_AR-cam	2003	3-1	Vilardaga et al., 2003
Signal transduction	Parathyroid hormone receptor	PTHR-cam	2003	3-1	Vilardaga et al., 2003
Signal transduction	Plasma membrane Calcium pump	BFP-PMCA-GFP	2007	3-1	Corradi and Adamo, 2007
Acetylation	Histone acetylation	Histac	2004, 2009	3-3	Lin et al., 2004; Sasaki et al., 2009
Lipid	Phosphatidylinositol (3,4,5)-trisphosphate (PIP3)	Fllip, FLIMPA	2003, 2013	3-4	Sato et al., 2003; Ueda and Hayashi, 2013
Lipid	Phosphatidylinositol (4,5)-bisphosphate (PIP2)	Pippi-PI(4,5)P_2_	2008	3-4	Nishioka et al., 2008
Lipid	Phosphatidylinositol (3,4)-bisphosphate (PI(3,4)P2)	Pippi-PI(3,4)P_2_	2008	3-4	Nishioka et al., 2008
Lipid	Phosphatidylinositol 4-phosphate (PI4P)	Pippi-PI(4)P	2008	3-4	Nishioka et al., 2008
Lipid	Phosphatidic acid	Pii	2010	3-4	Nishioka et al., 2010
Lipid	Diacylglycerol (DAG)	Daglas, DIGDA	2006, 2008	3-4	Sato et al., 2006a; Nishioka et al., 2008
Protein interaction	Actin		2004, 2008	2	Okamoto et al., 2004; Murakoshi et al., 2008
Protein interaction	PDK1-Akt interaction		2007	2	Calleja et al., 2007
Protein interaction	Protein tyrosine phosphatase 1B-receptor tyrosine kinases (PTP 1B-RTKs) interaction		2002	2	Haj et al., 2002
Protein interaction	Breast cancer resistance protein/ATP-binding cassette sub-family G member (BCRP/ABCG)		2010	2	Ni et al., 2010
Protein interaction	Cofilin-actin interaction		2008	2	Homma et al., 2008
Protein interaction	PTEN-Myosin V interaction		2009	2	van Diepen et al., 2009
Protease	Caspase-3	EGFP-DEVD-EBFP	1998	1	Xu et al., 1998
Protease	Caspase-8	CFP-c3-YFP-c6-mRFP	2002	1	Onuki et al., 2002
Protease	Caspase-9	SCAT9	2011	1	Joseph et al., 2011
Protease	Caspase-7	VDEVDc	2006	1	Li et al., 2006
Protease	Matrix Metalloproteinase (MMP)	YFP-MSS-CFP^*display*^, MTI-MMP-FRET biosensor	2007, 2008	1	Yang et al., 2007; Ouyang et al., 2008
Protease	Protease activity (Factor Xa)		1996	1	Mitra et al., 1996
Protease	Calpain activity	pYSCS	2000	1	Vanderklish et al., 2000
Protease	Presenilin	GFP-PSI-RFP	2009	3-1	Uemura et al., 2009
Other	Strain sensor	stFRET	2008	3-1	Meng et al., 2008
Other	Membrane potential	VSFP, Mermaid, ArcLight, VSFP-Butterfly	2001, 2008, 2012, 2013	3-1	Sakai et al., 2001; Tsutsui et al., 2008; Jin et al., 2012; Akemann et al., 2013
Other	Myosin II	GSldCB	1998, 2006	3-1	Suzuki et al., 1998; Zeng et al., 2006
Other	HIV Rev protein	YRGnC-11ad	2005	3-1	Endoh et al., 2005
Other	Redox	Redoxfluor, Gaskins	2010, 2011	3-1	Yano et al., 2010; Kolossov et al., 2011

The numbers in the Probe Design column correspond to the section number in the “Strategies of probe design” chapter of the main text. Names of probes are shown. See the webpage by Dr. Michiyuki Matsuda http://www.lif.kyoto-u.ac.jp/labs/fret/e-phogemon/unifret.htm for updated information.

Compared to small molecular weight fluorescent molecule-based FRET probes, genetically encoded FRET probes offer a number of advantages. They can be constructed easily with standard molecular biological techniques, thus making probe design simple and flexible. They can be expressed in cells by simply introducing vector DNA into neurons without protein purification and chemical labeling. Use of an appropriate DNA transduction method or a promoter to express the probe allow cell-type specific labeling. Due to these technical advantages, the genetically-encoded FRET probes are now widely used standard tools in biological systems.

### Strategies of probe design

**Figure 1:**
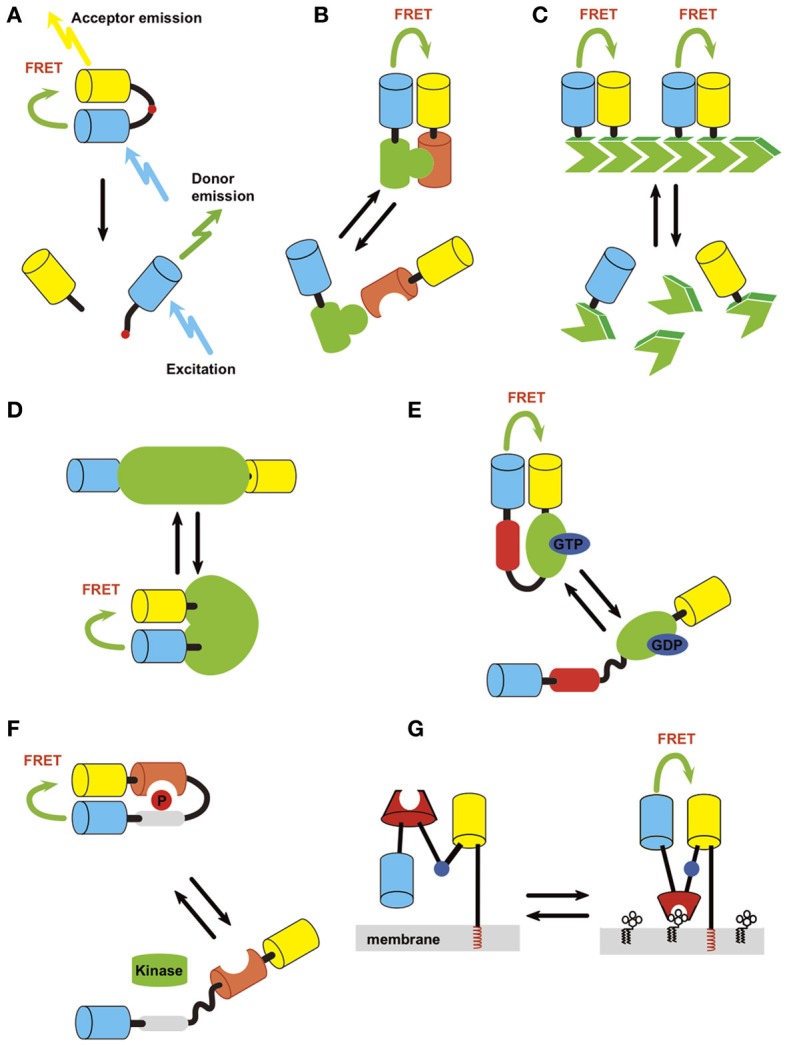
**Strategies of probe design**. Light blue, donor; yellow, acceptor. **(A)** Protease. **(B)** Intermolecular protein interaction. **(C)** Polymerization status. **(D)** Intrinsic conformation change of protein, which can be used to detect activation of a protein if it accompanies conformation change of the structure. **(E)** Conformation change of fusion protein induced by activation/inactivation. An example of detection of small GTPase activation (green) by small GTPase binding protein (red) is shown. **(F)** Conformation change of fusion protein induced by covalent modification/inactivation. Here an example of detection of kinase activity by substrate sequence (gray) and phosphoprotein binding domain (orange) is depicted. **(G)** Small molecule on membrane lipid.

Multiple genetically-encoded FRET probes have been developed for use in neuronal and non-neuronal cells. These probes can be classified into several categories depending on the approach used to detect different types of biological phenomena (Table 1, Figure 1).

#### Cleavage-based approach

The first reported GFP-based probe detecting Factor Xa activity employed the cleavage-based approach (Figure 1A) (Mitra et al., 1996). In this type of probe, a protease cleavage sequence was flanked by donor and acceptor fluorophores. Under basal conditions, FRET occurs between the fluorophores. However, cleavage of the target sequence causes a resultant separation of donor and acceptor molecules, leading to a decrease in FRET efficiency. The same approach was used to study other proteases including caspases (Xu et al., 1998; Onuki et al., 2002; Li et al., 2006; Joseph et al., 2011). One thing to note when using this type of probe is that the protease cleavage is irreversible. Therefore, it is not suitable for detecting a protease with high basal activity. Also, the measurement cannot be repeated multiple times as the uncleaved fraction decreases and the cleaved fraction accumulates over time.

#### Intermolecular FRET approach

The interaction between proteins can be monitored by intermolecular FRET, where one party of the protein complex is tagged by a donor and the other by an acceptor (Figure 1B). The interaction can be a heteromer of two different proteins or a homomer of the same protein. Application of this approach includes, small G-protein activity (Yasuda et al., 2006), 3-phosphoinositide-dependent protein kinase 1 (PDK)-Akt (Calleja et al., 2007), phosphatase and tensin homolog deleted from chromosome 10 (PTEN)-myosin V (van Diepen et al., 2009), and protein-tyrosine phosphatase 1B (PTP1B)-receptor tyrosine kinases (RTKs) (Haj et al., 2002) interaction (Table 1). A variant of this approach is homomultimer FRET where a monomer in a polymeric protein complex is labeled with both donor and acceptor molecules (Figure 1C), which allows the polymerization status of the protein to be monitored. This was employed to detect actin polymerization/depolymerization (Okamoto et al., 2004).

The quantitative aspect of FRET is difficult to control in intermolecular FRET (when compared with intramolecular FRET) because the expression level of donor and acceptor molecules often varies amongst cells. In contrast, in an intramolecular FRET probe, the donor and acceptor are on the same molecule and thus, the ratio of the donor to acceptor is always constant. Also, endogenous proteins may participate in forming protein complexes and this can decrease intermolecular FRET efficiency. Therefore, with intermolecular FRET, the efficiency must be measured as an average of multiples cells or compared before and after a treatment (e.g., induction of synaptic plasticity) in the same cell. In practice, a donor which does not interact with an acceptor increases the background of the measurement, whereas excess levels of acceptor molecules usually does not cause a problem (Okamoto and Hayashi, 2006). Therefore, whenever possible, excess acceptor molecules should be used.

#### Intramolecular FRET approach

This approach detects the conformational change of the probe via a change in the distance and angle of donor and acceptor proteins located on the same molecule. Because both fluorophores are on the same molecule, complications such as the differential redistribution of the donor and acceptor proteins and heterogeneity in the expression level of donor and acceptor among cells can be eliminated. Using this approach, many different probes have been generated to enable the detection of covalent modifications of proteins, membrane voltage, small biological molecules, and signal transduction (Table 1). One can design a probe to detect conformational change that is intrinsic to the protein of interest or design a fusion protein that changes its conformation upon the occurrence of a specified biological event. Advantage of intramolecular FRET is relative ease of constructing probe which shows FRET. But it is sometimes difficult to find right position of the fluorophore so that external stimuli change the FRET efficiency.

***Intrinsic conformation change of protein.*** If a protein of interest changes its conformation by activation/inactivation, one can design a probe to detect the conformational change as a way of monitoring the activity level (Figure 1D). This may be accomplished by flanking the protein with a donor and an acceptor or inserting one or both of the fluorophore(s) between the domains. This approach has been successfully employed for Ca^2+^/CaM-dependent protein kinase II (CaMKII) (Takao et al., 2005; Kwok et al., 2008; Fujii et al., 2013), calcineurin (Fujii et al., 2013), c-raf (Terai and Matsuda, 2005), p21 protein-activated kinase 1 (PAK1) (Parrini et al., 2009), B-raf (Terai and Matsuda, 2006), regulator of chromosome condensation 1 (RCC1) (Hao and Macara, 2008), vitamin A receptor (Shimozono et al., 2013) and to monitor changes in membrane potential (Tsutsui et al., 2008; Akemann et al., 2012). X-ray crystal structure is a useful guide to identify locations on a protein where the donor and acceptor pair can be placed.

***Conformation change induced by a specific protein interaction.*** Activation or inactivation of a protein can trigger an interaction with a specific target protein. By using such an interaction, one can design a FRET probe to detect the activation of a protein (Figure 1E). The cameleon probe mentioned above falls into this category. Another example is the Raichu series of probes that were developed to observe the activity of small G-proteins (Mochizuki et al., 2001). The basic structure of Raichu probes is comprised of four modules; a donor, an acceptor, a G-protein, and a G-protein-binding domain from its binding partner (Figure 1E). The inactive GDP-bound form does not interact with each other the G-protein-binding domain. Upon binding with GTP, the G-protein and G-protein-binding domain interact with each other to bring the two fluorophores into close proximity, thereby leading to FRET. This probe design strategy has been applied to Ras, Rho family protein, and other small G-proteins (Hao and Macara, 2008; Kiyokawa et al., 2011).

***Conformation change induced by a covalent modification of protein.*** This type of probe consists of a donor and an acceptor, which flank a substrate domain that can be covalently modified by the protein of interest and a protein domain that specifically recognizes the covalently modified protein (Figure 1F). When the protein is covalently modified, it binds to the adjacent recognition domain, leading to a conformational change in the entire molecule, resulting in a change in FRET. By making use of specific kinase substrate and phosphor-protein recognition domains, this strategy has been applied to the design of FRET sensors for kinases and phosphatases including PKA (Zhang et al., 2001), C (Violin et al., 2003), and D (Kunkel et al., 2007), Akt (Sasaki et al., 2003), and Src (Ting et al., 2001). It should be noted that this type of probe actually detects a temporal integration of both kinase and phosphatase activity. Also, there may be kinases or phosphatases other than the target protein, which also phosphorylate or dephosphorylate the probe.

***Small molecules on membranes.*** Using a similar strategy, small molecules on membranes can also be measured (Figure 1G). In this case, one of the fluorophores is tethered to the membrane through rigid α-helical linkers whereas the other fluorophore retains its flexibility via a gly-gly hinge. A specific lipid-binding domain is inserted in-between. When a small molecule binding domain interacts with its target of interest, a conformational change occurs through the hinge, resulting in an increase in FRET efficiency. This strategy has mainly been used to design probes for lipid second messengers such as phosphatidylinositol 3,4-bisphosphate (PI(3,4)P_2_), phosphatidylinositol 4,5-bisphosphate (PI(4,5)P_2_), phosphatidylinositol 3,4,5-trisphosphate (PIP_3_), phosphatidylinositol 4-monophosphate (PI(4)P), and diacylglycerol (DAG) (Sato et al., 2003, 2006a; Nishioka et al., 2008; Ueda and Hayashi, 2013).

### Detection of FRET

Several imaging methods for FRET detection are used in typical biological laboratory settings (Miyawaki, 2003; Yasuda, 2006, 2012).

#### Ratiometric FRET detection

In ratiometric FRET detection, the acceptor and donor images are acquired separately and the ratio of fluorescent intensity between the two images is subsequently calculated. When FRET occurs, the acceptor/donor ratio increases. Because any fluorescent microscopy (e. g., wide field, confocal, two-photon) can be used for this measurement, ratiometric FRET measurement is often used, though it is not best for several reasons. When performing this type of imaging, maximum care must be taken to minimize spectral bleed-through, to properly subtract background and to take into account the fluorophore relocalization. These factors make imaging in small structures particularly challenging. For example, CFP, a donor fluorophore that is often paired with YFP as an acceptor, can bleed into the YFP channel, thereby decreasing the signal/noise ratio. Hence to minimize bleed-through, a suitable band-pass filter should be used, even if the overall brightness of the signal is compromised. Also, background subtraction has to be performed with great care, as a subtle change in background can have a significant effect on the signal ratio. The issue of probe relocalization should also be carefully considered. This may be particularly problematic when measuring intermolecular FRET between two different molecules, which may differentially relocalize during neuronal plasticity. For example, if donor moves while the acceptor does not, it will cause an apparent change in fluorescent ratio without an actual change in protein interaction. This situation can be circumvented by using a probe with intramolecular FRET, where both donor and acceptor are on the same molecule or intermolecular FRET between homomers, where both are expected to move in parallel (Ni and Zhang, 2010). It is also possible to mathematically correct the FRET by separately measuring the amount of local acceptor. But in such cases, it is better to employ fluorescent life-time imaging microscopy, which relies only on donor fluorescence (see below).

#### Acceptor bleaching

When the acceptor is photobleached with an appropriate wavelength, the donor fluorescence is dequenched and increased. This maneuver, called acceptor bleaching, gives a quantitative reading of FRET as it depends only on the donor fluorescence intensity. Excitation light wavelength, intensity, and duration must be carefully chosen to photobleach only the acceptor fluorophore. The photobleaching of the donor fluorophore will underestimate the FRET. This can be done by simply illuminating the donor protein without an acceptor and making sure that donor fluorescence does not photobleach. It should be noted that the photobleaching of an acceptor is irreversible and therefore, acceptor photobleaching is a terminal experiment where only a single, specific and accurate static measure of FRET efficiency is needed (Miyawaki, 2003). Obviously, for this reason, acceptor bleaching is not compatible with time-lapse imaging.

#### Fluorescent lifetime imaging

The third approach to quantifying FRET relies on a parameter of fluorescence, called fluorescence lifetime (Yasuda, 2006). When a fluorescent molecule is excited, it emits fluorescence in a decaying manner from the time of activation, typically in exponential fashion. When FRET occurs, the donor fluoresecence lifetime is shortened. Because fluorescence lifetime is unaffected under a wide range of concentrations and does not depend on acceptor fluorescence, it is less prone to artifact caused by a change in the local concentration of donor and acceptor, which is especially important in heterooligomer FRET. In contrast, ratiometric measurement can show a pseudopositive signal caused by bleed-through between fluorescence channels, which can be an issue when measuring FRET from a structure where protein composition can change. Therefore, fluorescence lifetime imaging microscopy (FLIM) is the ideal choice for FRET detection.

There are largely two different methods of FLIM, time and frequency domain measurements (Yasuda, 2006). The time domain measures the fluorescence decay after a brief (< picoseconds) excitation pulse, while frequency domain measures lifetime by modulating the excitation light intensity and the detector gain differently (heterodyning) at high frequency (Yasuda, 2006). Both imaging systems are costly because FLIM requires a dedicated light-source and time-resolved detection. However, if one already has a two-photon microscope, adding components onto the existing system is straightforward. Current systems allow the detection of FRET signals at second order time resolution from single dendritic spines (Murakoshi et al., 2011), which is still slower than the ratiometric imaging that can go to video rate.

For the time domain measurement, time correlated single photon counting is currently widely used. This method measures the time elapses between an excitation pulse and an emitted single photon, which is binned into a histogram. The data will then be fitted to exponential curve (Yasuda, 2006). When two states are expected, such as in the case where both bound and unbound FRET pair coexist, it is possible to do double exponential fitting to obtain the ratio of two components (Yasuda, 2006). However, whether fitting double exponential is appropriate or not to a given FRET pair should be carefully considered based on the protein structure. For example, if donor forms a homodimer, it is enough to complicate the situation. When endogenous counterpart exists, often the case in a cell, the dimer can be either between two exogenous donor molecules or between one donor and one endogenous counterpart, in addition to the dimer made of two endogenous molecules. As a result, the acceptor interacts with either two, one or zero fluorescent molecules. Mathematically, it is possible to perform triple (or more) exponential fitting. However, such measurement requires (1) bright sample, (2) capability of hardware that captures high photon counts over a large number of pixels rapidly, and (3) ease of sophisticated data analysis. Cellular autofluorescence also complicates the analysis (Colyer et al., 2012). To circumvent this, one can calculate average lifetime of the photons, which theoretically gives lifetime in single exponential. This will not give absolute proportion of component showing FRET but by comparing the average lifetime over time, will give sufficient information even from a noisy decay curve not suitable for fitting (Lee et al., 2009; Murakoshi et al., 2011).

Another issue of the time domain measurement is the “dead zone” of the sampling. For example, in a system set up on a Ti-sapphire laser based two-photon microscope, the repetition rate of the laser is at 80 MHz or every 12.5 ns. There is always a dead zone of sampling between each cycle, where the acquisition system must reset for the next cycle. Given that many fluorescent proteins have lifetime of 2–5 ns range, the dead zone can limit the effective range of fitting and underestimate especially the component with longer lifetime. Recent studies that introduced widefield photon-counting detector and phasor analysis might provide a new approach to perform FLIM experiments, alleviating these shortfalls (Kwok et al., 2008; Colyer et al., 2012).

### Choice of fluorescence proteins

To effectively measure the change in the distance and angle between two fluorophores in a FRET construct, it is critical to start with a suitable pair of fluorescent molecules with efficient FRET. The efficiency of FRET (*E*) depends on several parameters characteristic to each pair of fluorescent proteins. Förster distance (*R*_0_), the distance at which the energy transfer efficiency is 50%, depends on the overlap of donor emission and acceptor excitation (*J*), quantum yield of the donor (*Q*_0_), and acceptor molar extinction coefficient (ε_*A*_). As the values for *J*, *Q*_0_, and ε_*A*_ increase, so does the value of *R*_0_, which in turn produces a larger *E* value. So far, CFP (or an improved version such as Cerulean or K26R/N164H mutant of ECFP) and YFP (such as Venus) is the most commonly for ratiometric FRET measurements. A CFP-YFP pair gives a *R*_0_ of 4.8–5.2 nm, depending on the variants used (Rizzo et al., 2006; Kwok et al., 2008; Lam et al., 2012). Recently, it was reported that the Clover and mRuby2 offers Förster radius of 6.3 nm and is currently considered to be the best FRET pair available to date (Lam et al., 2012).

For FLIM, enhanced GFP (EGFP) is often used as a donor, and paired with either monomeric red fluorescent protein (mRFP) or mCherry as an acceptor. The acceptor brightness is not an issue in FLIM as it relies solely on the donor fluorescence measurement. Therefore, non-fluorescent, quencher proteins such as REACh (Ganesan et al., 2006), darkVenus (Kwok et al., 2008), and super REACh (Lee et al., 2009) may also be used as acceptors to donor EGFP. Ideally, the donor should show a single lifetime with FLIM, which is the case for EGFP. The original enhanced CFP (ECFP) is not optimal as it shows two lifetime components, in addition to its relatively weak fluorescence. Cerulean and mTurquoise2 are both brighter and have mono exponential decay, therefore, can be used when the cyan range is needed (Rizzo et al., 2004; Goedhart et al., 2012).

EGFP has a weak tendency to dimerize (Zacharias et al., 2002), which can lead to issues with protein aggregation, depending on the protein it is fused with (Lantsman and Tombes, 2005). Therefore, monomerized versions of EGFP, such as the A206K mutant (the amino acid numbering is based on wild type GFP) is preferred for FRET experiments as it will reduce any pseudopositive FRET signal caused by non-specific aggregation. However, in certain cases, such as in cleavage-based protease sensors, the dimerization of donor and acceptor molecules can be beneficial to increase the difference in FRET efficiency before and after cleavage. In fact, a random mutagenesis study to enhance FRET efficiency of caspase probe lead to the identification of a CyPet-YPet pair (Nguyen and Daugherty, 2005), which was subsequently shown to form a dimer between donor and acceptor (Ohashi et al., 2007). For comprehensive review on fluorescence proteins, please refer to Shaner et al. (2005) and Newman et al. (2011).

### Application of FRET probes to study neuronal circuit dynamics

Numbers of FRET probes have been developed and tested in various cell types. Here we list some of the recent research accomplishments using FRET probes in neuronal circuits. See Table 1 for an extended list of various FRET probes.

#### Ca^2+^

Intracellular Ca^2+^ plays an important role in regulating various cellular functions such as signaling, gene regulation, cell death, and survival. Under basal conditions, the intracellular Ca^2+^ concentration is maintained at low levels by various Ca^2+^-extrusion and sequestration mechanisms. Upon neuronal activation, local intracellular Ca^2+^ concentration increases through influx from the extracellular fluid or efflux from the intracellular pool (Hayashi and Majewska, 2005). Different sources of Ca^2+^ can have distinct kinetics, subcellular localization and functions. Therefore, it is not very surprising that a Ca^2+^-sensing FRET probe was one of the first genetically encoded FRET sensors ever made (Miyawaki et al., 1997). A popular use of this type of probe is to detect neuronal circuit activity through a detection of action potentials as Ca^2+^ influx into cells via voltage dependent Ca^2+^ channels. The activity of hundreds of neurons can be simultaneously monitored (Wallace et al., 2008).

Since Miyawaki et al. characterized cameleon, the first Ca^2+^ sensing FRET probe, various probes with different affinities to Ca^2+^ have been reported (Miyawaki, 2005). Cameleon was expanded into the yellow cameleon series, which had greater sensitivity to Ca^2+^ and better signal/noise ratio (Nagai et al., 2004; Horikawa et al., 2010). Griesbeck et al. utilized troponin C and I to generate the Tn series Ca^2+^ sensor protein (Heim and Griesbeck, 2004). Cameleon has been mainly applied to zebrafish (Mizuno et al., 2013) and *C. elegans* (Haspel et al., 2010). Recently YC-Nano 140, new version of cameleon, was expressed to barrel cortex of mice using adeno-associated virus vector and showed different responses between two groups of neurons which are projected to different regions in neocortex (Chen et al., 2013).

Using a separate approach not involving FRET for its principle mode of detection, Nakai et al. generated G-CaMP (Nakai et al., 2001). G-CaMP was engineered to express CaM and a M13 peptide inserted in the β-barrel wall of GFP, which ultimately distorts its overall structure of GFP and quenches its fluorescence. An increase in Ca^2+^ concentration induces CaM and M13 peptide to interact, which then leads to a conformation change in the β-barrel. This in turn changes the protonation status of the fluorophore and dequenches the fluorescence. A related Ca^2+^ sensor termed pericam also utilizes a similar strategy (Nagai et al., 2001). Recently, B-GECO and R-GECO, a blue and red version of G-CaMP were developed to allow the simultaneous detection of calcium in more than one subcellular compartments or cell types (Zhao et al., 2011). With improvements in the sensitivity of probes and detection methods, it is now possible to visualize the Ca^2+^-influx in single dendritic spines evoked by unitary excitatory postsynaptic potential (epsp) (Ohkura et al., 2012). Currently G-CaMP is becoming the first choice for Ca^2+^ imaging, especially *in vivo* because it is convenient to detect the Ca^2+^ responses with one channel. However, a recent report comparing the sensitivity between G-CaMP3 and YCs in Purkinje cells of acute cerebellar slice from mice (Yamada et al., 2011) showed that YC exhibited better response than G-CaMP3, indicating that optimal probes need to be carefully chosen in a given brain region of interest.

#### A CaMKII activity sensor, Camui

CaMKII is a member of the serine/threonine protein kinase family that is highly expressed in the brain, especially at the postsynaptic density (PSD) of excitatory synapses (Kennedy et al., 1983; Chen et al., 2005). CaMKII has been highly implicated in both induction and maintenance of functional and structural LTP (Lisman et al., 2002; Matsuzaki et al., 2004). The activation of CaMKII precedes the structural enlargement of stimulated spines, suggesting that CaMKII is a molecular trigger of downstream processes that lead to structural changes. In addition, the CaMKII has structural role at the synapse through its capacity to bundle F-actin (Okamoto et al., 2007, 2009).

Under basal conditions, CaMKII is kept inactive by intrasubunit steric block of the substrate-binding site (S site) in the kinase domain by a pseudosubstrate region within the autoinhibitory domain (Lisman et al., 2002). Binding of Ca^2+^/CaM to the regulatory domain (adjacent to the autoinhibitory domain) alters its conformation and disrupts the inhibitory interaction at the S site. This disruption releases the kinase domain from autoinhibition and allows it to rapidly self-phosphorylate threonine 286 (T286) of CaMKII, as well as other substrates. CaMKII autophosphorylation at T286 prevents the autoinhibitory domain from binding with the T site of the catalytic domain and from blocking the kinase activity, thereby allowing the kinase to retain substantial activity even in the absence of Ca^2+^. Thus, this holoenzyme remains active for a prolonged period of time, significantly outlasting that of a Ca^2+^ spike. Based on these observations, CaMKII was proposed as a memory molecule, which can be used to store long term information after a synapse undergoes LTP (Lisman et al., 2002).

However, direct demonstration of the persistent activation of CaMKII after the induction of LTP was lacking because of a deficiency in effective methods to detect the spatial and temporal activation of CaMKII at the single spine level. To circumvent this, a FRET probe, Camui, was engineered by employing the intramolecular FRET approach to detect the conformational change associated with CaMKII activation by fusing donor and acceptor fluorophores to both termini of CaMKII (Takao et al., 2005; Kwok et al., 2008). Camui shows FRET in its basal inactive state. Addition of ATP, CaM, and Ca^2+^ leads to a rapid and persistent decrease in FRET. The conformational change due to binding of Ca^2+^/CaM and autophosphorylation is accountable for the change in FRET. This persistent, Ca^2+^-independent change in FRET is absent when ATP is omitted or when a kinase dead mutant is used. Furthermore, a phosphoblocking mutant (T286A) stops the persistent change in FRET, whereas a phosphomimicking mutant (T286D) shows decreased FRET without Ca^2+^ stimulation. Hence, Camui detects the collective activation of CaMKII by the binding of Ca^2+^/CaM and the autophosphorylation at T286. Using a FLIM version of Camui, green-Camuiα, Lee et al. discovered that CaMKII activity is only transient (<2 min) after the induction of structural LTP (sLTP) even though CaMKII activation is required for sustaining structural synaptic plasticity. This is much shorter than what had been believed (Lee et al., 2009).

**Figure 2:**
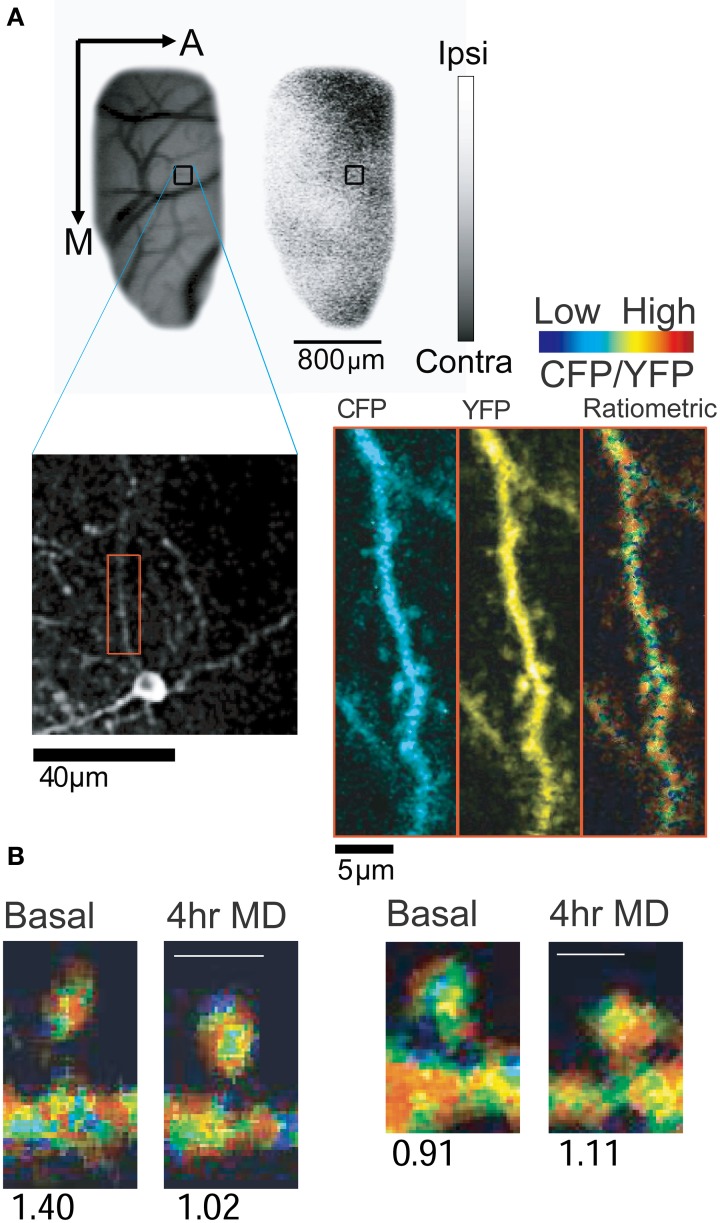
***In vivo* imaging of CaMKII activity using Camui. (A)** Expression of Camui in ferret visual cortex allowed for visualization of CaMKII activity in dendrites and spines of a neuron in a specific ocular dominance (OD) domain. Blood vessel and OD maps were acquired using intrinsic signal optical imaging (*Upper panel*: A, anterior; M, medial). Gray scale indicates ocular dominance index (white, ipsilateral eye dominated; black, contralateral eye dominated). Blood vessels map and the low magnification two-photon microscopic image were cross referenced to identify the two-photon images (*Lower panel*) in a given OD map. A dendritic segment (red box) is magnified (*Right*) and displayed as channel separated images (CFP and YFP) as well as a ratiometric image in intensity-modulated display mode, indicating the CFP/YFP ratio. Warm hue represents high CaMKII activity. **(B)** Sample images of spines with decreased (left) or increased (right) CaMKII activity after 4 h of monocular deprivation. Numbers below the images indicate the normalized CFP/YFP ratio, a measurement of FRET signal. From Mower et al. (2011).

We investigated the spatial and temporal regulation of CaMKII in rapid ocular dominance (OD) plasticity in layer II/III of ferret visual cortex *in vivo*, a paradigmatic model for studying the role of sensory experience in shaping cortical neural circuits (Mower et al., 2011). By taking advantage of the superficial location of layer II/III pyramidal neurons for optical detection of Camui signals (Figure 2A), we found that brief monocular deprivation (MD, 4 h) leads to activation of CaMKII at most synapses in the deprived eye domains (Figure 2B). However, a change in CaMKII activity was not observed in the spines located in binocular and non-deprived eye domains following the same visual manipulation. Four hours of MD also lead to the elimination of a small fraction of spines in the deprived eye domain, whose basal CaMKII activity was lower than the average CaMKII activity in the same cortical site. The spines that persisted after MD had either high basal CaMKII activity or increased activity. Therefore, the emerging picture of the role of CaMKII activity *in vivo* is that (1) the eliminated spines have low CaMKII activity (although not all spines with low activity are removed) and (2) high CaMKII activity might have a protective role for spines and these preserved spines could potentially serve as a substrate for the reorganization of intracortical presynaptic partners.

At first, this result is seemingly at odds with the study by Lee et al., where they observed a transient activation of CaMKII by LTP induction with glutamate uncaging. However, this result most likely reflects the ability of CaMKII to respond to different neuronal activity patterns (De Koninck and Schulman, 1998; Fujii et al., 2013). In the study by Lee et al. (2009), CaMKII is activated by local *N*-methyl-D-aspartate type glutamate receptor (NMDA-R) activation. However, in the visual cortex, it likely detects an integration of complex local and global activity patterns that encompass both Hebbian and homeostatic mechanisms. Further studies are required to fully elucidate the role of CaMKII in synaptic plasticity both *in vitro* and *in vivo*.

#### Small G-protein

Small G-protein family, including Ras, Rho, Ran, Rab, Sar/Arf subfamilies, is a large group of signaling molecules that control various cellular functions (Saneyoshi and Hayashi, 2012). The activity of small G-protein is controlled by intrinsic GTPase activity and by the type of guanine nucleotide it is bound with. GTP-bound form consists active form, which is converted into GDP-bound form by the GTPase activity. The cycle between GDP-bound inactive and GTP-bound active forms is regulated by three classes of proteins, guanine nucleotide exchange factors (GEFs), GTPase-activating proteins (GAPs), and guanine nucleotide dissociation inhibitors (GDIs) (Saneyoshi and Hayashi, 2012). GEFs exchange GDP bound on a small G-protein with GTP, which leads to an activation of signaling activity of the small G-protein. The GTP-bound forms of G-protein lapse into inactive forms when GAP induces activation of GTPase activity that converts the bound GTP to GDP. GDI removes GDP-bound inactive forms of G-proteins from cell membranes and therefore maintains them in inactive forms.

Two family member of Ras family, Ras itself and Rap are implicated in synaptic plasticity. Zhu et al. showed that Ras relays the NMDA-R and CaMKII signaling that drives synaptic delivery of α-amino-3-hydroxy-5-methyl-4-isoxazolepropionic acid type glutamate receptors (AMPA-Rs) during LTP (Zhu et al., 2002). In contrast, Rap mediates NMDA-R-dependent removal of synaptic AMPA-Rs that occurs during LTD. Thus, Ras and Rap serve as independent regulators for potentiating and depressing central synapses. Ras is also implicated in spine formation. The expression of a constitutively active Ras in neocortex neurons lead to an increase in spine density (Gartner et al., 2005). Conversely, a loss of SynGAP, a Ras-GAP that expresses specifically in the brain, leads to an increase in spine formation and enlargement of spine size (Vazquez et al., 2004).

In order to elucidate the Ras activity during LTP in the spines of hippocampal neurons, Yasuda et al. designed an intermolecular FLIM-based probe to detect Ras activity, in which momomeric EGFP was tagged to the N-terminus of Ras, and two momomeric RFPs were attached to the N- and C-termini of the Ras binding domain (RBD) of Raf (Yasuda et al., 2006). When Ras at the plasma membrane is activated, RBD is recruited to the membrane and binds to Ras, resulting in an increase in FRET. Using this probe, they investigated the activity of G-proteins in single dendritic spines in CA1 pyramidal neurons during sLTP. After the induction of sLTP, Ras was activated, which was then maintained for 30 min (Yasuda et al., 2006). Interestingly, the Ras signaling is not restricted to spines but spreads over 10 μm into dendritic shafts and eventually reaches neighboring spines, which can subsequently undergo sLTP with only weak stimulation (a stimulation that would normally induce only temporary potentiation) (Harvey et al., 2008b). These data suggest that the spread of Ras-dependent signaling is necessary for the local regulation of the LTP induction threshold.

Rho family G-proteins, including ras homolog family member (Rho), ras-related C3 botulinum toxin substrate (Rac), and cell division control protein 42 homolog (Cdc42), are small GTP binding proteins that control the actin cytoskeleton (Komatsu et al., 2011; Saneyoshi and Hayashi, 2012). Because actin is the major cytoskeletal protein in dendritic spines, the role of the Rho family G-proteins on the maintenance and rearrangement of spine morphology has been investigated (Saneyoshi and Hayashi, 2012). The expression of a constitutively active form of Rac1 in hippocampal pyramidal neurons leads to an increase in the number (Tashiro et al., 2000), length and width of spines (Zhang and Macara, 2006), while a dominant negative had the opposite effect (Nakayama et al., 2000; Zhang and Macara, 2006; Impey et al., 2010). In contrast, a constitutively active form of RhoA reduces the density of spines (Tashiro et al., 2000; Impey et al., 2010) and causes a simplification of dendritic branch pattern (Nakayama et al., 2000). Inhibition of RhoA activity leads to an increase in the number of spines in some neurons (Tashiro et al., 2000; Impey et al., 2010). Cdc42 is also implicated in spine morphogenesis (Tashiro et al., 2000; Irie and Yamaguchi, 2002).

Murakoshi also applied the same Ras probe design strategy to construct probes for Rho family protein (Murakoshi et al., 2011). The temporal and spatial extent of activity spreading over the dendritic shaft was investigated (Murakoshi et al., 2011). Activity of both RhoA and Cdc42 was maintained for up to 30 min, which is consistent with the observation that the filamentous (F-) actin/globular (G-) actin equilibrium moves toward F-actin after LTP induction (Okamoto et al., 2004, see below). RhoA spreads with a length constant of 4.5 μm along the dendrite. On the other hand, Cdc42 activity was restricted only in the stimulated spine, whose length constant is 1.9 μm.

#### Phosphatidylinositol 3,4,5-trisphosphate (PIP_3_)

PIP_3_ is a phosphoinositide that plays an important role in a variety of cellular functions. PIP_3_ is produced from phosphatidylinositol 4,5-bisphosphate (PIP_2_) by phosphoinositide 3-kinase (PI3K) in response to hormone and neurotransmitter while PTEN converts PIP_3_ back to PIP_2_. In hippocampal pyramidal neurons, PIP_3_ is crucial for maintaining AMPA-R clustering during LTP (Arendt et al., 2010). PIP_3_ also regulates neuronal polarity, dendritic arborization, and nerve growth factor-induced axonal filopodia formation (Jaworski et al., 2005; Ketschek and Gallo, 2010). In order to exert these functions, local PIP_3_ accumulation leads to the recruitment of effector proteins such as Akt (Thomas et al., 2001), WASP family Verprolin-homologous protein (WAVE) (Oikawa et al., 2004) and GEF of small G proteins to specific subcellular compartments (Han et al., 1998; Shinohara et al., 2002; Innocenti et al., 2003).

In order to investigate PIP_3_ function and regulation in spines, we developed a FLIM-based PIP_3_ FRET probe, FLIMPA3, by concatenating a donor, a specific PIP_3_-binding domain, flexible di-glycine hinge, and an acceptor tethered to the membranes through rigid α-helical linkers (Sato et al., 2003; Murakoshi et al., 2008; Ueda and Hayashi, 2013) (Figure 1G). When FLIMPA3 was expressed in hippocampal CA1 pyramidal neurons, we found that PIP_3_ showed greater accumulation in spines than in dendritic shafts under basal conditions (Ueda and Hayashi, 2013). PI3K inhibitor treatment decreased PIP_3_ accumulation in spines, indicating that PIP_3_ accumulation is largely due to basal PI3K activity in spines. This result is consistent with a previous report in which PI3K is ubiquitously localized in neuronal cells, but only becomes active after AMPA-R binding (Man et al., 2003). During sLTP, PIP_3_ in spines was reduced. Application of a PTEN inhibitor did not significantly change the reduction in PIP_3_. Additionally, the reduction of PIP_3_ after sLTP was highly correlated with PIP_3_ enrichment before sLTP induction. Therefore, the reduction in PIP_3_ during sLTP is likely to be due to the addition of membrane from the dendritic shaft. Interestingly, whilst PIP_3_ globally decreases in spines during sLTP, we observed a specific accumulation of PIP_3_ in spinules, filopodia-like protrusions found on spines. When PIP_3_ in spinules was blocked by a PI3K inhibitor that reduces PIP_3_ levels, the number of spinules after sLTP were diminished, indicating that PIP_3_ in spinules regulates spinule formation.

Electron microscopic studies found that spinules could be trans-synaptically endocytosed by presynaptic terminals as separate vesicles from the postsynaptic side (Spacek and Harris, 2004). Therefore, the trans-endocytosis of spinules may serve as a mechanism for retrograde signaling or may aid postsynaptic membrane remodeling by removing excess membrane (Spacek and Harris, 2004). Accumulated PIP_3_ in spinules that traffic to the presynaptic side may act as a retrograde signal or contribute to the formation of new synapses with functional presynaptic boutons.

#### Extracellular signal-regulated kinase (ERK)

ERK is a serine/threonine protein kinase that belongs to the mitogen-activated protein kinase (MAPK) family, which plays important roles in a variety of cellular functions such as cell differentiation, proliferation, and survival (Chang and Karin, 2001). In neuronal circuits, ERK is involved in a wide range of functions including the regulation of dendritic protein synthesis (Impey et al., 1998a,b; Roberson et al., 1999; Davis et al., 2000; Patterson et al., 2001; Waltereit et al., 2001), morphological changes in dendritic spines (Wu et al., 2001; Goldin and Segal, 2003) and hippocampal LTP and memory formation *in vivo* (Giovannini et al., 2001). Abnormal ERK signaling is associated with mental retardation (Costa et al., 2002).

In order to obtain information about the spatiotemporal dynamics of ERK activity in neuronal cells, several FRET-based probes have been developed. Miu2 detects the conformational change of ERK activation by flanking ERK with CFP and YFP (Fujioka et al., 2006). Erkus is based on the detection of substrate protein phosphorylation (Sato et al., 2007) (Figure 1F). The ERK substrate sequence was obtained from EGFR and fused to the phospho-binding domain from FHA2 by a flexible peptide linker. The D domain, a sequence that selectively binds to ERK was attached to increase the specificity and efficiency of phosphorylation. This fusion protein was flanked by CFP and YFP. When phospho-substrate peptide is phosphorylated by active ERK, the phosphoprotein-binding domain interacts with the phospho-substrate peptide, leading to a change in overall conformation, which can be detected by a change in FRET efficiency. EKAR uses a similar approach but with a different substrate and a phosphoprotein-binding domain (Harvey et al., 2008a).

Using EKAR in hippocampal pyramidal neurons, Harvey et al. observed ERK activity induced by back-propagating action potentials (Harvey et al., 2008a). Stimulated bursts of action potentials caused global Ca^2+^ influx through voltage-gated Ca^2+^ channels, leading to Ras activation, an upstream molecule of ERK (Yasuda et al., 2006; Harvey et al., 2008b). After stimulation, ERK activity reached a peak by around 5 min, then gradually decreased, and finally returned to basal levels by 30 min. The time course of ERK activation was longer than that of Ras, consistent with the idea that ERK is the downstream effector of Ras. They also investigated ERK activity in the somatic cytoplasm and nucleus of neuronal cells. After theta-burst stimulation, ERK activity in both regions was up-regulated in a parallel manner, indicating that global Ca^2+^ influx through VGCCs can diffuse rapidly between these two compartments (Harvey et al., 2008a).

#### Chloride sensor

Cl^−^ ion regulates neuronal properties such as intracellular pH, cell volume, and fluid secretion (Duran et al., 2010). More importantly, Cl^−^ is a major carrier of electrical current in inhibitory synaptic transmission mediated by GABA and glycine receptors. The basal level of intracellular chloride ions (Cl^−^) is maintained by a number of mechanisms including chloride transporter system that consist of Na^+^-Cl^−^, Na^+^-K^+^-2Cl^−^, and K^+^-Cl^−^ transporters, and the activation of tonic GABA receptors, calcium-activated Cl^−^ channels, cAMP-activated Cl^−^ channels, cell-volume regulated anion channels, and transporters localized within subcellular organelles (Duran et al., 2010). Since all these factors sum up to determine the intracellular Cl^−^ concentration, it is of a great interest to visualize the dynamics of intracellular Cl^−^.

The chloride sensor, Clomeleon, consists of CFP, a flexible peptide linker, and a Cl^−^ sensitive YFP (with S65G, S72A, K79R, T203Y, H231L mutations) (Kuner and Augustine, 2000). YFP intensity is quenched in the presence of Cl^−^, thereby changing FRET efficiency in a Cl^−^ concentration-dependent manner. Using this probe, in hippocampal dissociated cultures of neurons and glial cells, the developmental time course of Cl^−^ concentration was investigated (Kuner and Augustine, 2000). While the Cl^−^ concentration in glia cells was low throughout embryonic and postnatal stages, the concentration in neurons was higher at embryonic stages, and then decreased during postnatal development, consistent with the observation that activation of GABA receptors in immature neurons leads to neuronal excitation rather than inhibition (Kuner and Augustine, 2000). Using this probe, it was also possible to observe Cl^−^ influx through GABA receptors in hippocampal CA1 pyramidal neurons following interneuron stimulation (Berglund et al., 2006). However, at this point, the sensitivity of the Cl^−^ sensor is not as good as to visualize Cl^−^ influx induced by unitary inhibitory postsynaptic current (ipsc). This would require further elaboration of the probe.

#### Actin

Actin is the major cytoskeletal protein in dendritic spines (Matus, 2005; Okamoto et al., 2009). It exists in equilibrium between two forms, globular (G-actin) and filamentous actin (F-actin) (Okamoto et al., 2009; Saneyoshi and Hayashi, 2012). Actin has a rapid turnover time within the dendritic spine. An experiment using fluorescence recovery after photobleaching (FRAP) of GFP-fused actin revealed that over 85% of actin in dendritic spines is dynamically turning over, with an average time constant of 44 s (Star et al., 2002). This dynamic turnover is the underlying molecular basis of motility and morphological changes of spines (Okamoto et al., 2004, 2009; Matus, 2005; Honkura et al., 2008).

As in non-neuronal cells, F-actin in dendritic spines undergoes a unique directional treadmilling as revealed with experiments using a photoactivatable (PA)-GFP-actin or a photoconvertable fluorescent protein (Honkura et al., 2008; Frost et al., 2010). G-actin is added to the barbed end of F-actin at the periphery of dendritic spines and at the base of the dendritic spine, F-actin is continuously disassembled to G-actin at the pointed end of actin. Taken together, there is an overall directional movement of F-actin from the periphery toward the spine base (Honkura et al., 2008; Frost et al., 2010). Another way to look at this is to divide the actin population into different pools. The first pool of F-actin, found at the periphery, has a relatively high turnover of about 40 s (Honkura et al., 2008). The second pool is the population that resides at the base of spines, with a turnover time of 17 min (Honkura et al., 2008). These two pools are relatively static and help to maintain the overall spine shape and size. In addition, there is a third pool that appears after LTP induction (Honkura et al., 2008). The turnover time of this pool is 2–15 min and it spreads all over the spine. This pool is required to maintain dendritic spine enlargement upon sLTP induction. If this pool extrudes into the dendritic shafts then sLTP was not maintained.

**Figure 3:**
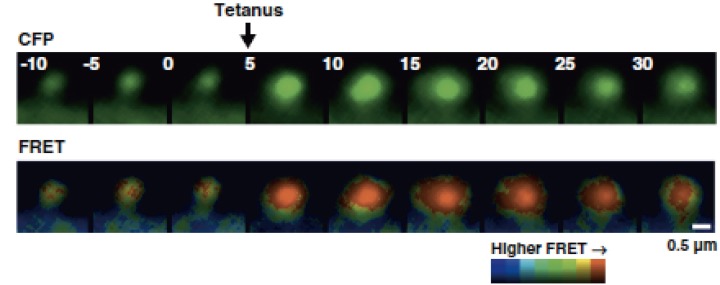
**Dendritic spine expressing actin FRET probe**. Single dendritic spine that was subjected to local tetanic stimulation is accompanied by the enlargement of spine and actin polymerization. From Okamoto et al. (2004).

Actin exists in equilibrium between F-actin/G-actin but it was not known how the F-actin/ G-actin equilibrium changes during synaptic plasticity. This is because the dendritic spine is too small and does not show discrete F-actin structure that is observable with light microscopy. To circumvent this, an intermolecular FRET approach was used to monitor the F-actin/ G-actin equilibrium (Okamoto et al., 2004). The distance between actin monomers in F-actin is 55 Å, which is within the appropriate range to be detected with FRET. Actin was tagged with CFP and YFP as a donor and an acceptor, respectively. Using this approach, Okamoto et al. observed actin dynamics in hippocampal CA1 pyramidal neuronal cells during bidirectional plasticity (Okamoto et al., 2004). Upon tetanic stimulation, the equilibrium of F-actin/G-actin shifted toward F-actin, which was accompanied by spine enlargement (Figure 3). In contrast, prolonged low-frequency stimulation, typically inducing LTD, lead to spine shrinkage and actin depolymerization. This evidence suggests that the equilibrium of F-actin/ G-actin regulates bidirectional structural plasticity.

#### Voltage sensors

Electrophysiological recordings are considered to be the “gold standard” technique for measuring neuronal membrane potentials. However, several drawbacks to this method exist, such as the invasive nature of the technique and limitations in the number of neurons that can be measured simultaneously. To circumvent these issues, small molecular weight voltage-sensitive fluorescent dyes have been used with some success. The main disadvantage of using small molecular weight voltage-sensitive fluorescent dyes is the lack of cell-type specificity, because the dyes are generally bogus loaded and taken up by cells in a non-specific manner. It is also important to note that the dyes can diminish over time or cause toxicity. Therefore, these dyes are mostly suited for use in acute experiments.

Genetically-encoded membrane potential sensors, VSFP2 (Sakai et al., 2001) and Mermaid (Tsutsui et al., 2008), allow us to visualize the membrane voltage of a large number of individual neurons with high temporal resolution. Both probes are based on a membrane embedded phosphatase that senses voltage, Ci-VSP, a protein derived from tunicate, *Ciona intestinalis*. Ci-VSP is composed of a voltage-sensor domain (VSD) and phosphatase domain (Murata et al., 2005). The phosphatase domain on the C-terminus was replaced with a fluorophore pair fused in tandem. Membrane depolarization causes a conformational change in the overall structure, leading to a decrease in the distance between fluorophores, and ultimately a change in FRET efficiency. In cultured cortical neurons expressing Mermaid, a stimulated burst (30 pulses at 100 Hz) of spikes could be observed (Tsutsui et al., 2008). Recently, VSFP-butterfly and ArcLight were developed, where the acceptor was moved from the C-terminus to the N-terminus (Akemann et al., 2012, 2013; Jin et al., 2012). VSFP-butterfly has been used to visualize changes in membrane voltage elicited by the stimulation of a single whisker in layer 2/3 pyramidal neurons in the mouse barrel cortex (Akemann et al., 2012). The authors also succeeded in visualizing spontaneous slow brain oscillations traveling over the somatosensory cortex (Akemann et al., 2012).

## Concluding remarks

In 1990s, the readout of synaptic plasticity was mostly limited to the size of electrical response of synapse. The data were analyzed by applying to mathematical model of synaptic transmission established from studies on neuromuscular junction, which later turned out to be not compatible to the central synapse and caused a huge confusion in the field. The title of a review written by Sanes and Lichtman “Can molecules explain long-term potentiation?” (Sanes and Lichtman, 1999), well represents the sentiment around that time on the never-ending debate on the mechanism of LTP. Fortunately, the recent introduction of technologies to optically measure the activity of molecules involved in synaptic plasticity has drastically changed the field and successfully clarified a number of points that remained unsolved before and provided new concepts of synaptic plasticity.

In the quest to understand the molecular mechanisms underpinning neuronal circuit plasticity, FRET has played a critical role in revealing important insights into the spatiotemporal dynamics of the key players. However, limiting its further application, it has been empirically known that it is difficult to establish transgenic mice expressing FRET probes (Hara et al., 2004). This may be due to the repeat of very similar DNA sequence (CFP and YFP) within transgene (Kamioka et al., 2012). It is also possible that probe proteins work as a gain-of-function mutant that hampers the function of endogenous proteins (Hara et al., 2004). Nonetheless, number of transgenic animals expressing FRET probes has been increasing (Hara et al., 2004; Berglund et al., 2006; Zhang et al., 2010; Yamaguchi et al., 2011; Kamioka et al., 2012; Wang et al., 2012; Thunemann et al., 2013). Additional difficulty lies in the detection of FRET, especially in the *in vivo* preparation. In practice, the animal's heartbeat and breathing introduce not only the movement of the cells during imaging but also the hemodynamic noise and therefore changes the absorbance in the optical path of the excitation and emission of fluorescence (Akemann et al., 2012). This will affect the accuracy of FRET data acquisition. With continued technological advances, it will be possible to apply FRET to increasingly complex preparations, even *in vivo*, to fully understand the complicated neuronal signaling processes that occur in the ever-changing brain.

## Author contributions

Yoshibumi Ueda, Showming Kwok, and Yasunori Hayashi jointly wrote the manuscript.

### Conflict of interest statement

Yasunori Hayashi is partly supported by Takeda Pharmaceuticals Co. Ltd. and Fujitsu Laboratories. The other authors declare that the research was conducted in the absence of any commercial or financial relationships that could be construed as a potential conflict of interest.
